# Controlling soil disturbance of a lunar regolith simulant bed during depressurization in a vacuum chamber

**DOI:** 10.1038/s41598-021-81317-1

**Published:** 2021-01-21

**Authors:** Gyu-Hyun Go, Jangguen Lee, Taeil Chung, Byung Hyun Ryu, Hyunwoo Jin, Li Zhuang, Hyu Soung Shin, Jae Hyun Kim, Tae Sup Yun

**Affiliations:** 1 Department of Civil Engineering, Kumoh National Institute of Technology, Gumi, Republic of Korea; 2 Department of Future Technology and Convergence Research, Korea Institute of Civil Engineering and Building Technology (KICT), Goyang, Republic of Korea; 3 Department of Civil Engineering, Kangwon National University, Chuncheon, Republic of Korea; 4 School of Civil and Environmental Engineering, Yonsei University, Seoul, Republic of Korea

**Keywords:** Engineering, Physics

## Abstract

A dusty thermal vacuum chamber (DTVC) containing a regolith simulant bed is essential for testing equipment and techniques related to lunar surface exploration. Space agencies have been reluctant to operate a DTVC because of the challenge of controlling soil disturbance of the lunar regolith simulant bed during pumping down or depressurization, which may contaminate or even damage the chamber and vacuum equipment. There appears to be no previously available solution to this problem, or how to avoid it. We investigated the mechanism of soil disturbance during depressurization and established a criterion for evaluating its occurrence. The proposed criterion was validated by extensive experiments and numerical modelling to simulate air evacuation from soil voids. There is a critical pressure difference (CPD) between the top and bottom of the lunar regolith simulant bed that causes soil disturbance during depressurization. We found a simple equation estimating the CPD and further provided guideline on the optimum depressurization rate to avoid soil disturbance before the target vacuum level is achieved under varying soil conditions.

## Introduction

Lunar exploration is shifting focus from remote sensing using orbiters to surface missions with rovers or landers^1,2^. In 2009, NASA announced preliminary results from the Lunar Crater Observation and Sensing Satellite (LCROSS) impact mission, confirming the presence of ice on the Moon^3^. Since then, interest in the in situ resource utilization (ISRU) such as lunar subsurface exploration and related studies has been increasing^4–9^. A thermal vacuum chamber containing a regolith simulant bed—a dusty thermal vacuum chamber (DTVC)—is necessary for the testing and verification of equipment for a lunar surface mission^10–14^. Various vacuum chambers have been developed for testing equipment and drilling performance with lunar regolith dust, which is one of the highest-risk factors in such a testing^15–20^. Operation of a DTVC containing a regolith simulant bed involves the challenge of dust contamination in a vacuum chamber because soil disturbs in pumping down process. For example, Kleinhenz and Wilkinson reported vacuum pump malfunction due to dust and dust resistant pump was used^5^. Kleinhenz mentioned difficulties to maintain the initial soil condition for drill testing during pumping down^10^. Zhang et al. designed a special regolith container with small holes in the container wall to prevent soil disturbance^16^. Chung et al. created vacuum environment without soil disturbance by adjusting the pumping down speed^18^. From previous studies, soil disturbance in the vacuum chamber causes contamination of vacuum chamber, damage of pumping system and devices, and changes in initial condition of soil bed, which are unfavourable for performance testing of other equipment on the soil bed. Therefore, one of the critical issues with a DTVC operation is to prevent soil disturbance during the pumping down process. Although there have been several trials reporting mitigation of soil disturbance in the vacuum chamber^5,16,18^, quantitative evaluation on the soil disturbance during the pumping down process and guide to prevent the occurrence of soil disturbance have been rarely reported.

The pumping is usually described by a depressurization rate (d*P/*d*t*), defined as the decrease in the air pressure in the chamber per unit time. During this process, air pressure differences arise within and outside the regolith simulant bed in the chamber. If this pressure difference is high enough the simulant bed will be disturbed, and this is usually displayed as cracking, boiling in the bed, or even dust emission involving the ejection of fine soil particles.

**Figure 1:**
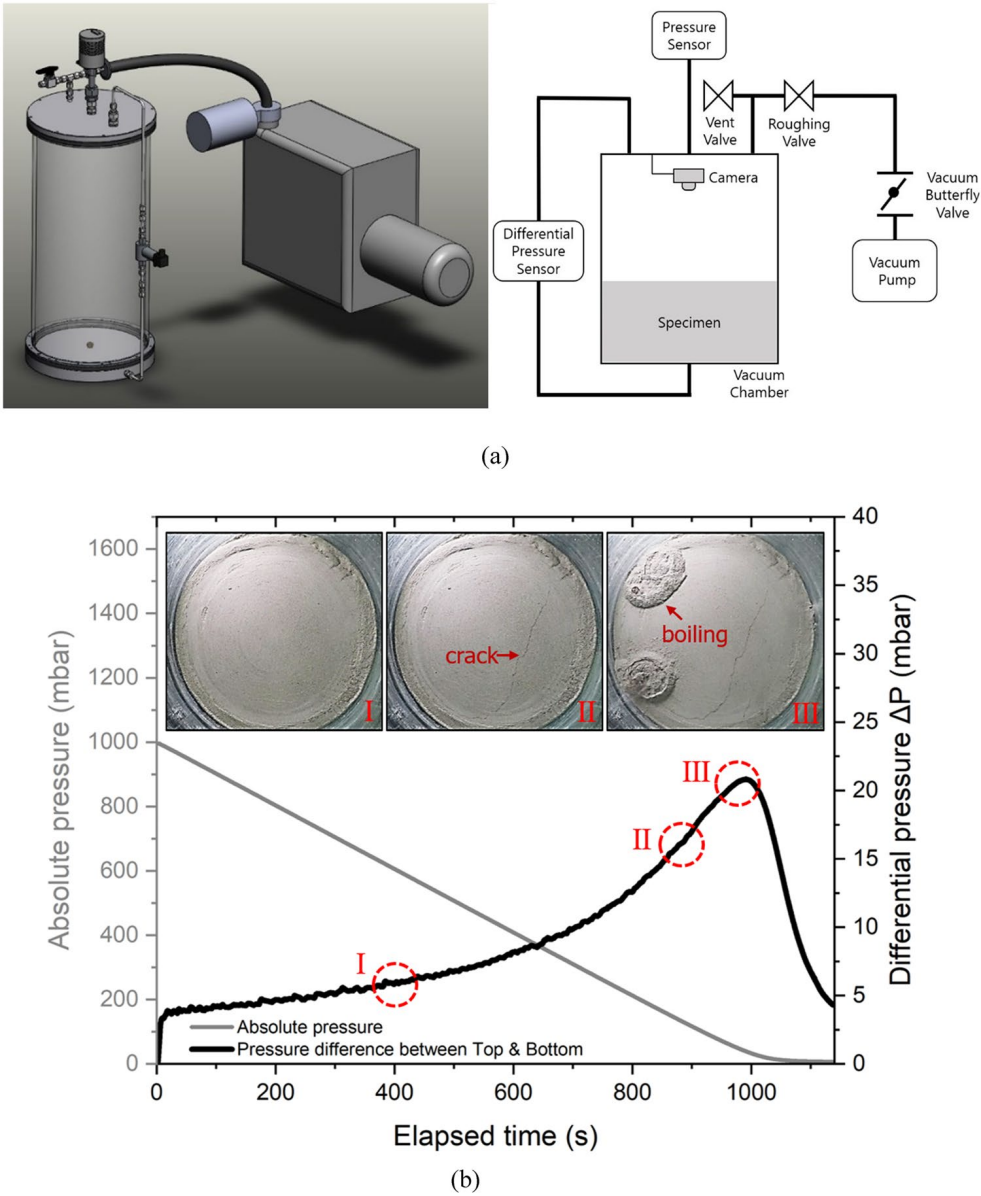
Laboratory experiments for the evaluation of soil disturbance. (**a**) Diagrams of the laboratory apparatus used. (**b**) Occurrence process of soil disturbance during the depressurization at a specific condition. The absolute pressure decreases while the differential pressure increases with elapsed time. In this case soil disturbance initiated at *t* = 882 s.

In this study, we investigated the soil disturbance phenomenon through laboratory experiments (Fig. 1a), and a criterion for soil disturbance initiation was proposed. Numerical modelling was applied to validate the proposed criterion and for further investigation of the main influencing parameters. Optimal depressurization rates corresponding to different conditions of soil bed were suggested to avoid soil disturbance during depressurization and therefore to maintain the initial conditions of the bed.

## Results

### Depressurization test and numerical simulation

**Figure 2:**
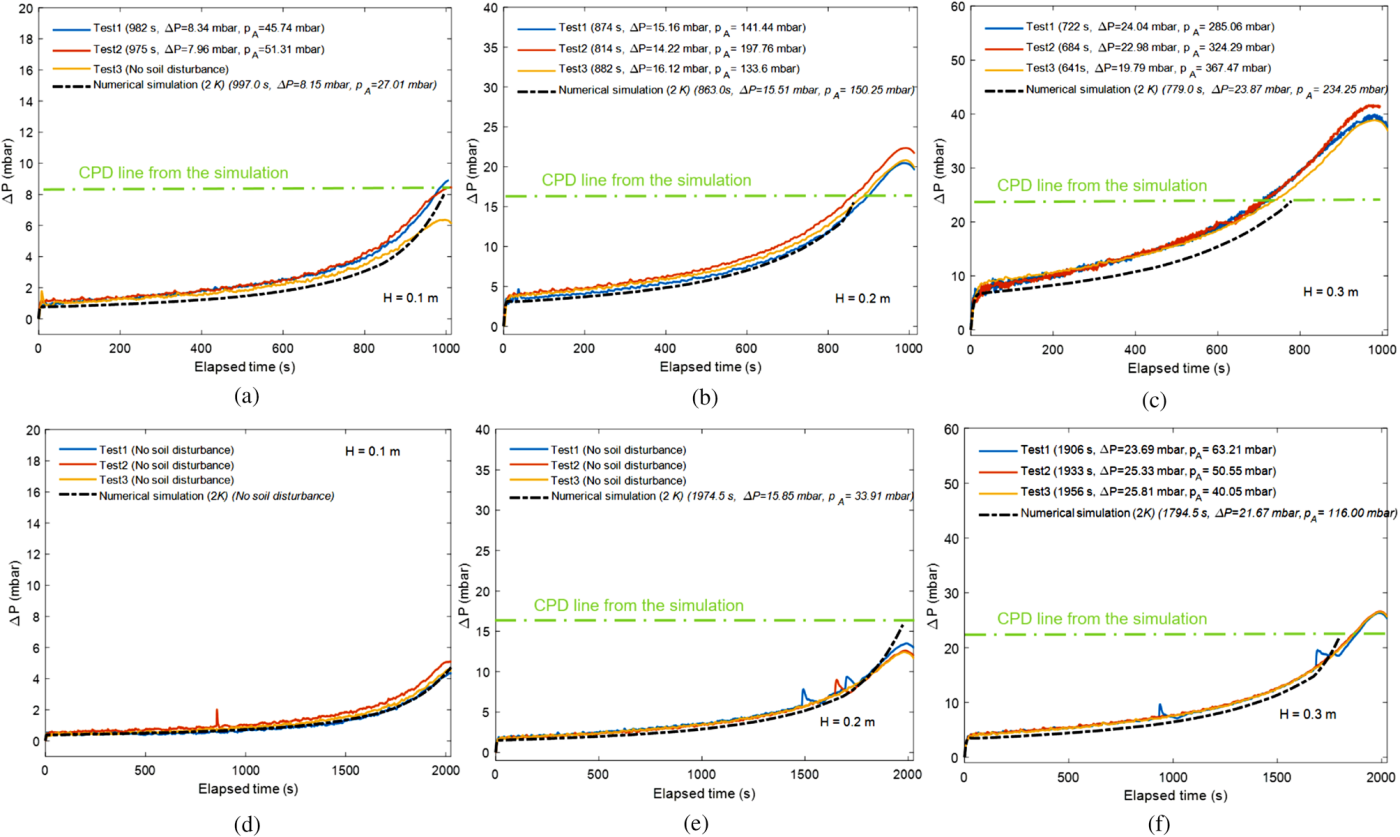
Comparison of experiments and predictions. (**a**–**c**) Results for a depressurization rate of 1.0 mbar s^–1^. (**d**–**f**) Results for a depressurization rate of 0.5 mbar s^–1^. *H* : height of the simulant bed in both experiments and numerical simulations. *CPD* : Critical Pressure Difference; ΔP is calculated as the difference between the pressure measurements at the top and bottom of the simulant bed.

The disturbance of a lunar regolith simulant bed during depressurization process in a cylindrical chamber is illustrated in Fig. 1b. The pressure difference, Δ*P*, was measured between the top and bottom of the bed, and increased during evacuation. Cracking on the bed surface at a certain Δ*P* indicated the initiation of disturbance; Δ*P* continued to increase until regolith simulant boiling occurs, and the resulting dust, comprising disturbed simulant particles. Through these tests, we figured out that there was a threshold pressure difference between the bed top and bottom causing the soil disturbance during depressurization. The soil disturbance occurred only if the threshold value was met. We defined the threshold as the ‘critical pressure difference’ (CPD). Thus, we established a novel control criterion for preventing soil disturbance while achieving a target vacuum level within an acceptable time. Numerical modelling including the soil disturbance criterion was evaluated by comparing simulated and experimental measurements. Experiments were performed with three target heights, *H*, of a simulant bed (0.1, 0.2, and 0.3 m) at three depressurization rates (0.25, 0.5, and 1.0 mbar s^–1^). Experimental results and simulation results are compared in Fig. 2, where the time of first occurrence of soil disturbance and the corresponding differential pressure Δ*P* are noted in each subfigure. Simulation results show excellent agreement with experimental measurements in both the time and the Δ*P* at the first occurrence of soil disturbance, which always initiates at the surface of the simulant bed. The results under the depressurization rate of 0.25 mbar s^–1^ were not given in Fig. 2 because no soil disturbance occurred in either the laboratory experiments or the numerical analysis (Supplementary Table S2). This also means that a constant depressurization rate of 0.25 mbar s^–1^ is sufficiently safe though it may not be the optimum.

### Estimation of CPD

**Figure 3:**
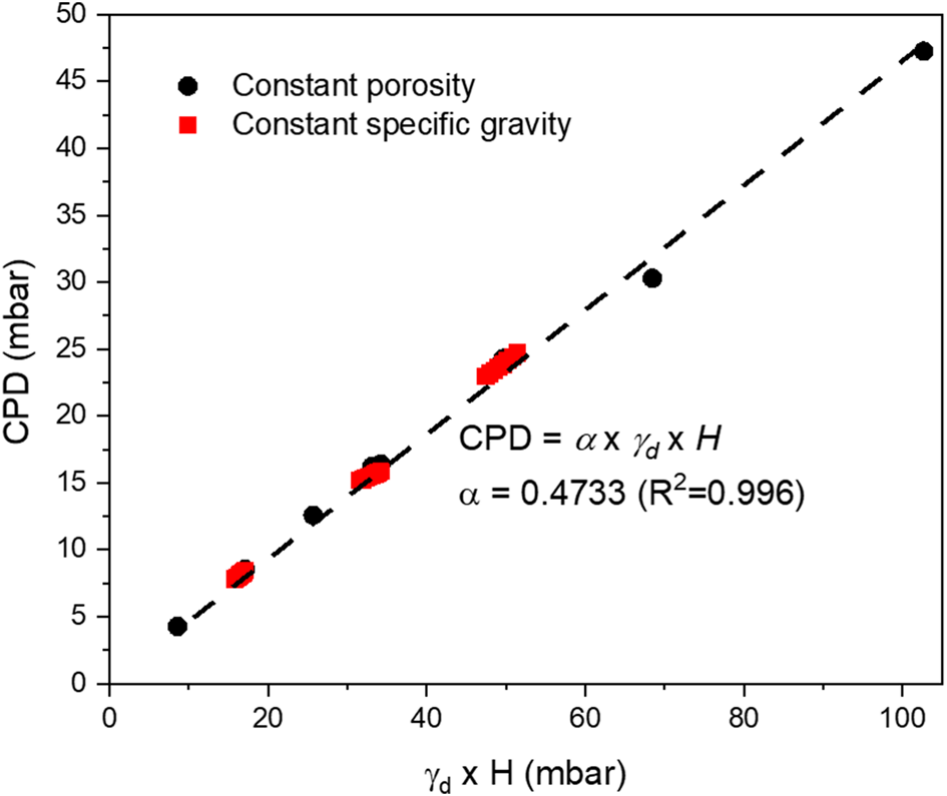
CPD versus overburden pressure at the bottom of the bed (γ_d_ · *H*). γ_d_ is the dry unit weight of soil, and *H* is the height of the simulant bed.

For similar bed heights (e.g., Fig. 2b,e, Fig. 2c,f), the pressure differences between the top and bottom of the bed at the initiation of disturbance are always very close, regardless of the depressurization rate or input value of intrinsic permeability. This indicates that there is a threshold value of pressure difference that causes soil disturbance. Through numerical modelling, we investigated CPD at a variety of conditions by varying the three main influencing parameters, including the specific gravity (*G*_s_ = 1.5, 2.9, or 6.0), simulant bed height (*H* = 0.1, 0.2, or 0.3 m), and material porosity (*n* = 0.367–0.438). The specific gravity for soils is defined as the ratio of the unit weight of soil particles to the unit weight of water (*γ*_*w*_), usually at 4 °C. The porosity is determined by soil particle size distribution as well as the packing density (loose or dense). It can be estimated from the specific gravity and the dry unit weight of the soil (*γ*_*d*_). We found that the CPD has an approximately linear relationship with vertical overburden pressure at the bottom of the bed caused by simulant weight, as shown in Fig. 3. Therefore, CPD can be estimated using Eq. (1):

1 $${\text{CPD}}\, = \,\alpha \cdot \gamma_{d} \cdot H$$

2 $$\gamma_{d} \, = \,Gs \cdot \gamma_{w} \cdot \left( {1 - n} \right)$$

where *α* is estimated to be ~ 0.47 based on data obtained from numerical simulations (Fig. 3).

## Discussion

Occurrence of soil disturbance is related to the height of simulant bed, hydraulic properties of simulant, and the depressurization rate, and its accurate prediction required an understanding of its underlying mechanism. As indicated in Fig. 2, when the pressure difference exceeds the CPD, the time of occurrence of soil disturbance is influenced mainly by the depressurization rate. The higher the depressurization rate, the earlier soil disturbance occurs. Moreover, as the intrinsic permeability increases, the occurrence of soil disturbance is delayed because the pressure difference increases slowly. Our findings imply that soil disturbance during the depressurization of a DTVC can be avoided by real-time monitoring and management of the differential pressure between the top and bottom of the simulant bed.

**Figure 4:**
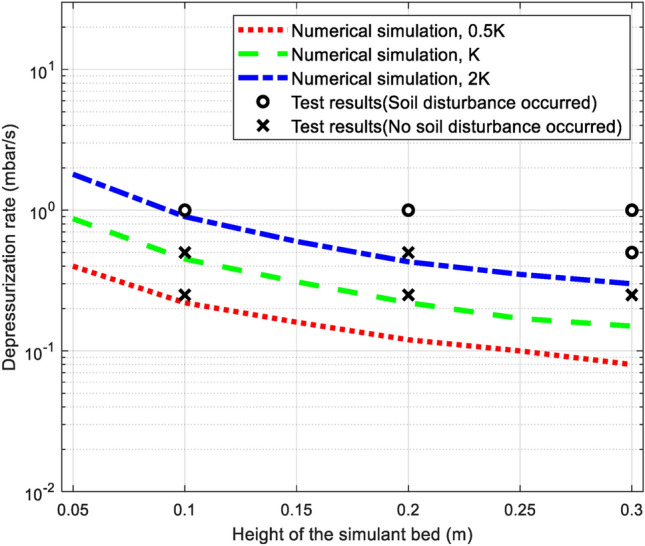
Maximum allowable depressurization rate for different simulant bed heights; the three curves correspond to different intrinsic permeabilities of 0.5* K*, *K*, and 2* K*. *K* = 2.55 × 10^−13^ m^2^.

Managing the pressure difference would rely on controlling the rate of depressurization. For the design of the chamber used here, Fig. 4 compares the calculated maximum allowable depressurization rates for various bed heights (*H*) and intrinsic permeability (*K*) with experimental results. As has been pointed out in Fig. 2, numerical results using the intrinsic permeability of 2* K* are in good agreement with experimental measurements on the evolution of pressure difference during the pumping process. Therefore, the simulation results with 2* K* in Fig. 4 provide relatively more reasonable guidance to determine the maximum depressurization rate. By applying the optimal depressurization rate presented here, the initial conditions of the simulant bed can be maintained without disturbance during depressurization and ensure the safe operation of the vacuum chamber within an acceptable time frame.

## Methods

### Laboratory experiments

Experimental study on fluidization of fine particles at reduced pressure or vacuum conditions based on laboratory experiments using cylindrical chambers has been reported in previous study^21–23^. A cylindrical acrylic chamber with an inner diameter of 280 mm and height of 600 mm was fabricated for the verification of the depressurization simulation model (Fig. 1a). The lid of the chamber is made in aluminium. A differential pressure sensor is installed at the center of a pipe that connects two holes being fabricated at the top and bottom of the chamber, for measuring the differential pressure between the top and bottom of the simulant bed during the test. To prevent the bottom hole from being clogged with soil particles, a 10 mm-thick microporous ceramic filter and a paper filter are used. A camera was installed below the lid of the chamber to record images of the bed surface during a test. An oil rotary vane vacuum pump having pumping capacity of 1000 L/min and a throttle valve with 32 mm inner diameter were used for air depressurization. A capacitance diaphragm gauge having measurement range of 0.1 mbar to 1100 mbar with 0.5% accuracy was used to measure the absolute pressure in the chamber. Depressurization rate was controlled by adjusting both pumping speed and throttle valve opening. The starting output of the pump was set at 15% of the maximum pumping speed and it was linearly increased to 100% until that the absolute pressure was reduced to 500 mbar. The main purpose of this operation is to prevent the depressurization rate being too high at the initial stage of pumping. Measurement value of depressurization rate was sent to a control system and it will be either decreased or increased to match a given target value by controlling of the throttle valve opening automatically.

### Simulant bed preparation

The lunar regolith simulant used in this study was the Korean Lunar Simulant Type 1 (KLS-1) passed #200 sieve with particle size ranging 0.002–0.075 mm. The KLS-1 has a very similar particle size distribution (PSD) to that of the JSC-1 and for details about the physical and mechanical properties we refer to Ryu et al.^24^. The PSD is one of the most significant factors determining physical properties of the soil including the intrinsic permeability. In this study, the soil bed is composed of pure fines having relatively low intrinsic permeability (2.55 × 10^−13^ m^2^) compared to that of the regolith simulant, e.g., 1 × 10^−12^ to 6.1 × 10^−12^ m^2^ corresponding to the bulk density range of 1550 to 2000 kg m^−3^ reported for the JSC-1A lunar regolith simulant^25^. According to the research findings (Fig. 4), a larger depressurization rate is allowed for a higher intrinsic permeability of the simulant bed. Therefore, the conservative conditions were considered in the laboratory experiments. In addition, the KLS-1 simulant has an apparent cohesion of around 1.85 kPa as measured from the direct shear test^24^. This could explain why the soil disturbance was initiated in the pattern of a crack, as shown in Fig. 1b. To achieve similar dry unit weights between experiments, simulant samples had similar heights and weights in the vacuum chamber, as hydraulic conductivity is highly dependent on the void ratio^26–28^. The sample should also have uniform density throughout its depth. A miniature cone penetrometer of 10 mm diameter was used to verify the uniformity of the regolith^29^. To maintain similar initial conditions of the simulant bed for different tests, the following preparation method was developed: (1) stir the sample with a mixer (Supplementary Fig. S1a); (2) remove the mixer and fix the sample container to a shaker table to homogenize the sample by vibration (Supplementary Fig. S1b); (3) level the surface of the specimen and check its height; and (4) conduct the cone penetration test (CPT) at a penetration rate of 1.5 mm s^–1^ (Supplementary Fig. S1c). Uniformity and consistency of regolith samples were confirmed by the CPT results, after which the sample was again stirred with a soil mixer and conditioned with a shaker table. Finally, the vacuum test was performed. The initial conditions of the specimens for each test are listed in Supplementary Table S1, showing the similar initial specimen setups. To obtain the representability of the experiments, each test was conducted three times under the same conditions. The experimental results for each test represented great representability showing a small error. The reason why such a high experiment reproducibility could be made is that particular attention had been paid to maintain the initial conditions in the sample preparation (Supplementary Fig. S1).

### Numerical validation method

We present a numerical model to simulate depressurization by applying air-phase seepage theory. Poroelasticity theory proposed by Biot^30,31^ was applied in the numerical modelling, and the model was implemented using the commercial finite element analysis software, Comsol Multiphysics (Version 5.4). The domain was considered as a porous medium comprising a solid matrix with pores, and with fluid flow in the pores. The deformation of the solid matrix was negligible, and the intrinsic permeability of the medium was assumed to be constant. The equations used in the model include force-equilibrium and mass-balance equations for the fluid.

3 $$\nabla \cdot \sigma = \nabla \cdot (C:\varepsilon - \alpha_{B} Ip_{f} ) = 0$$

4 $$\zeta = \alpha_{B} \nabla \cdot u + \frac{{p_{f} }}{M}$$

where *σ* represents the total stress (kPa), *C* is the elastic tensor of the solid matrix, *ε* is the strain tensor, *α*_*B*_ is the Biot constant representing the coupling between stress and pore pressure, *I* is the identity matrix, *p*_*f*_ is pore pressure (kPa), *ζ* is the increment of fluid content, *u* is the displacement of the solid matrix. 1/*M* is related to the compressibility of the fluid and solid matrix and is defined as:

5 $$\frac{1}{M} = \frac{{\alpha_{B} - n}}{{K_{s} }} + \frac{n}{{K_{f} }}$$

where *K*_*s*_ is the bulk modulus of the solid matrix (Pa), *K*_*f*_ is the bulk modulus of the fluid (Pa), and *n* is porosity. If the compressibility of the solid-grain material is negligible compared to that of the drained bulk material (i.e., *K*_*s*_ = ∞), Eq. (4) can be simplified to:

6 $$\frac{1}{M} \approx \frac{n}{{K_{f} }} = nc_{f}$$

where *c*_*f*_ is the compressibility of fluid (Pa^–1^).

The flow of a fluid in porous medium can be described by Darcy’s law. Darcy's law which supposes a laminar flow is valid for Reynolds number less than 1, but the upper limit can be extended up to 10^32^. In this study, the upward seepage air flow during depressurization apparently was examined to be within a linear laminar flow regime. Thus, the mass flow rate (*q*), and can be expressed as

7 $$q = - \rho_{f} \frac{K}{\mu } \cdot \nabla p_{f}$$

where *ρ*_*f*_ is the density of the fluid (kg m^–3^), *K* is the intrinsic permeability (m^2^), and *μ* is dynamic viscosity (Pa s). The intrinsic permeability *K* is a critical input parameter for the simulation, and is determined by the particle-size distribution and packing density of the regolith simulant. Here, it was back-calculated from hydraulic conductivity, *k* (m s^−1^)

8 $$K=\frac{\mu }{{\rho }_{f}g}k$$

In this study, the hydraulic conductivity of the regolith simulant was measured in accordance with the standard test method for measurement of hydraulic conductivity (ASTM D5856, 2015)^33^, and *k* = 2.44 × 10^−6^ m s^−1^. The intrinsic permeability was therefore determined to be *K* = 2.55 × 10^−13^ m^2^.

Numerical simulations used three different values of intrinsic permeability, being 0.5, 1.0, and 2.0 times of the calculated intrinsic permeability given above. The reason why the three cases were considered in the simulation is that there exists an error range for the measured value of hydraulic conductivity. It should be also noted that the intrinsic permeability considered in this study have the same order of magnitude of 1 × 10^−13^ m^2^.

As there is a relationship between increments in fluid content and the mass flow rate (Eq. 9), the mass-balance equation is summarized as Eq. (10):

9 $$\frac{\partial \zeta }{{\partial t}} = - \frac{1}{{\rho_{f} }}\nabla \cdot q$$

10 $$\rho_{f} (\frac{{\alpha_{B} - n}}{{K_{s} }} + \frac{n}{{K_{f} }})\frac{{\partial p_{f} }}{\partial t} - \nabla \cdot (\rho_{f} \frac{K}{\mu }\nabla p_{f} ) = - \rho_{f} \alpha_{B} \frac{\partial \varepsilon }{{\partial t}}$$

The numerical simulation thus evaluates the poro-elastic behaviour of the soil medium through the coupled analysis of the force equilibrium (Eq. 3) and mass equilibrium (Eq. 10).

If air flow is considered in a porous medium, it can be assumed that the fluid follows the ideal gas equation. Change of air density during depressurization is described as:

11 $$\rho_{a} = \frac{{\omega_{a} }}{RT}p_{a}$$

where *ω*_*a*_ is the molecular mass of air (kg kmol^–1^), *R* is the universal gas constant (8.31432 J (mol K)^–1^), *T* is absolute temperature (K), and *p*_*a*_ is absolute pressure (kPa). Therefore, the mass-balance equation for the air in soft soil (*α*_*B*_ = 1) is given by

12 $$\rho_{a} \left( {nc_{a} } \right)\frac{{\partial p_{a} }}{\partial t} - \nabla \left[ {D_{d}^{*} \nabla p_{a} } \right] = - \rho_{a} \frac{\partial \varepsilon }{{\partial t}}$$

where ${D}_{d}^{*}$ is the coefficient of transmission of the air phase, and can be represented as ${D}_{d}^{*}$= *ρ*_*a*_*K*/ *μ*_*a*_, where *μ*_*a*_ is the dynamic viscosity of air (Pa s), *c*_*a*_ is compressibility of air (Pa^–1^), and *p*_*a*_ is the air pressure (Pa). Under isothermal conditions, the compressibility of air can be expressed as^34^:

13 $$c_{a} = \frac{1}{{K_{f} }} = \frac{1}{{p_{a} }}$$

Depressurization began at the top of the soil bed, and the other boundary conditions (including the bed bottom) were defined as “undrained” conditions. For the mechanical part, the top boundary was set to “free”, the bottom was defined as “fixed”. Both wall boundaries were defined as “roller”, which allows the vertical direction movement (Supplementary Fig. S2). Based on the aforementioned methodology, this study conducted numerical simulations to evaluate the air flow behaviour of soil specimen during the depressurization process (Supplementary Fig. S3).

### Soil disturbance criterion

Soil bed is disturbed due to an upward air flow during depressurization. We propose a criterion on soil disturbance initiation being when the upward differential pressure (Δ*p*) overcomes the soil weight for a given unit depth:

14 $$\Delta p\, \ge \,\rho_{{\text{d}}} \cdot g \cdot \, \Delta h$$

Δ*h* is the vertical depth difference between the two points, *g* is gravitational acceleration (m s^−2^), and *ρ*_*d*_ is soil dry density. The occurrence of soil disturbance is determined by the criterion in the Eq. (14) while the time of occurrence is dependent on the intrinsic permeability and the depressurization rate. In the numerical modelling based on finite element method, each simulation was terminated once the condition in the Eq. (14) was met in any soil element.

## Supplementary Information

Supplementary Information

## Data Availability

All data used in this paper are available upon request.

## Code availability

The code that supports the findings of this study is available from the corresponding author upon request.
