# Supplementary material for: Controlling soil disturbance of a lunar regolith simulant bed during depressurization in a vacuum chamber
Source: Sci Rep. 2021 Jan 21;11:1878. doi: 10.1038/s41598-021-81317-1 (PMC7820600; doi:10.1038/s41598-021-81317-1)
Supplement: Supplementary file 1 — Supplementary Information [file 41598_2021_81317_MOESM1_ESM.docx]

Supplementary Information

**Controlling soil disturbance of a lunar regolith simulant bed during depressurization in a vacuum chamber**

Gyu-Hyun Go, Jangguen Lee^*^, Taeil Chung, Byung Hyun Ryu, Hyunwoo Jin, Li Zhuang,

Hyu Soung Shin, Jae Hyun Kim, and Tae Sup Yun

^1^Department of Civil Engineering, Kumoh National Institute of Technology, Republic of Korea

^2^Department of Future Technology and Convergence Research, Korea Institute of Civil Engineering and Building Technology (KICT), Republic of Korea

^3^Department of Infrastructure Safety Research, KICT, Republic of Korea (Present affiliation: Kangwon National University)

^4^ School of Civil and Environmental Engineering, Yonsei University, Republic of Korea

*Corresponding author, e-mail: jlee@kict.re.kr

| Target height of simulant bed (m) | Depressurization rate  (mbar s^–1^) | Average measured value of three repeated tests | | | | |
| --- | --- | --- | --- | --- | --- | --- |
|  |  | Regolith mass  (kg) | Measured simulant bed  height (m) | Dry unit weight  (kg m^–3^) | Void ratio | Porosity |
| 0.1 | 0.25 | 10.67 | - | - | - | - |
|  | 0.5 |  | 0.106 | 1635 | 0.798 | 0.444 |
|  | 1.0 |  | 0.106 | 1634 | 0.800 | 0.444 |
| 0.2 | 0.25 | 21.34 | - | - | - | - |
|  | 0.5 |  | 0.205 | 1693 | 0.737 | 0.424 |
|  | 1.0 |  | 0.206 | 1685 | 0.745 | 0.427 |
| 0.3 | 0.25 | 32.01 | 0.305 | 1705 | 0.724 | 0.420 |
|  | 0.5 |  | 0.306 | 1697 | 0.733 | 0.423 |
|  | 1.0 |  | 0.302 | 1719 | 0.710 | 0.415 |

Supplementary Table S1. Initial conditions of the tested simulant bed in laboratory experiments.

Supplementary Table S2. Soil disturbance evaluation results in laboratory tests and numerical simulations.

| Soil bed height | d*P*/d*t*  0.25 mbar s^–1^ | | d*P*/d*t*  0.5 mbar s^–1^ | | d*P*/d*t*  1.0 mbar s^–1^ | | CPD^**^ |
| --- | --- | --- | --- | --- | --- | --- | --- |
|  | Test | Simulation | Test | Simulation | Test | Simulation |  |
| 0.1 m | X | X | X | X | O(^*^978.5s) | O(997.0s) | 8.15 mbar |
| 0.2 m | X | X | X | O(1974.5s) | O(^*^856.7s) | O(863.0s) | 16.0 mbar |
| 0.3 m | X | X | O(^*^1931.7s) | O(1794.5s) | O(^*^682.3s) | O(779.0s) | 23.9 mbar |

^*^ mean value of the time of occurrence

^**^ critical pressure difference

| 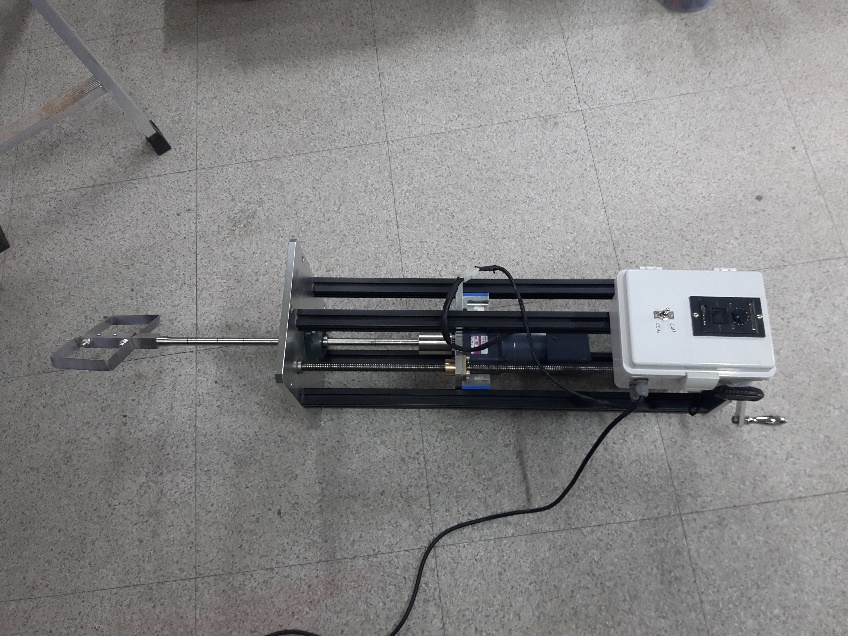 | 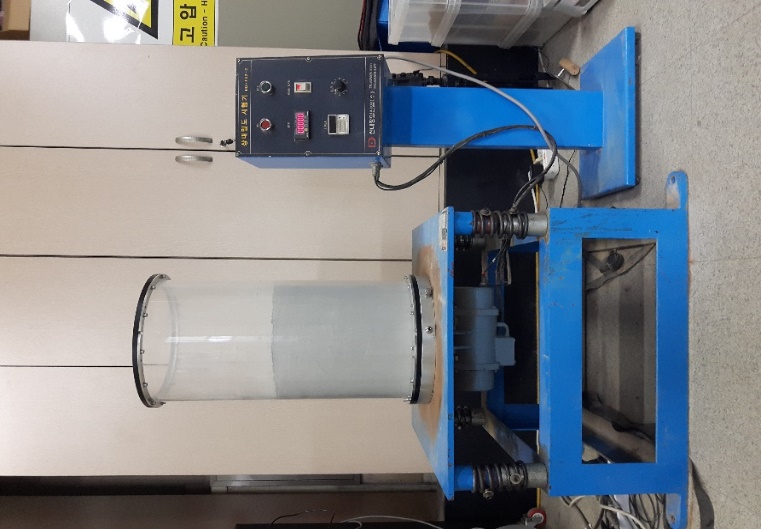 | 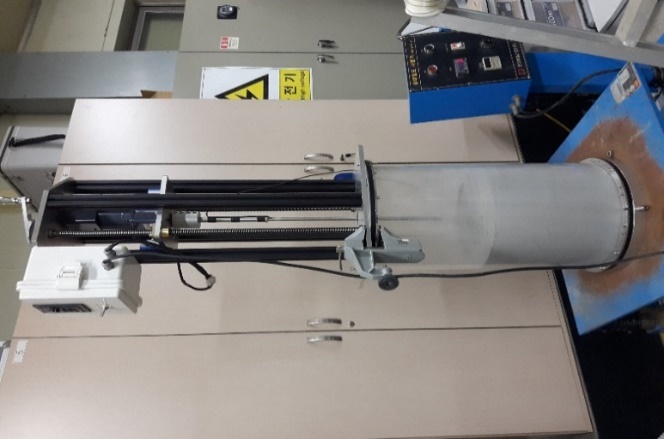 |  |
| --- | --- | --- | --- |
| (a) | (b) | (c) |  |
| ****  (d) | | | |

**Supplementary Fig. S1.** Preparation of a regolith simulant bed and cone penetrometer test (CPT) results. (a) A soil mixer used to stir the regolith sample; (b) soil container fixed on a shaker table; (c) Conduct CPT to check uniformity and consistency of the simulant bed; (d) CPT results for different simulant beds with a height (H) of 0.1 m, 0.2 m and 0.3 m. Diameter of the cone was 10 mm and the penetration speed was 1.50 mm/s.


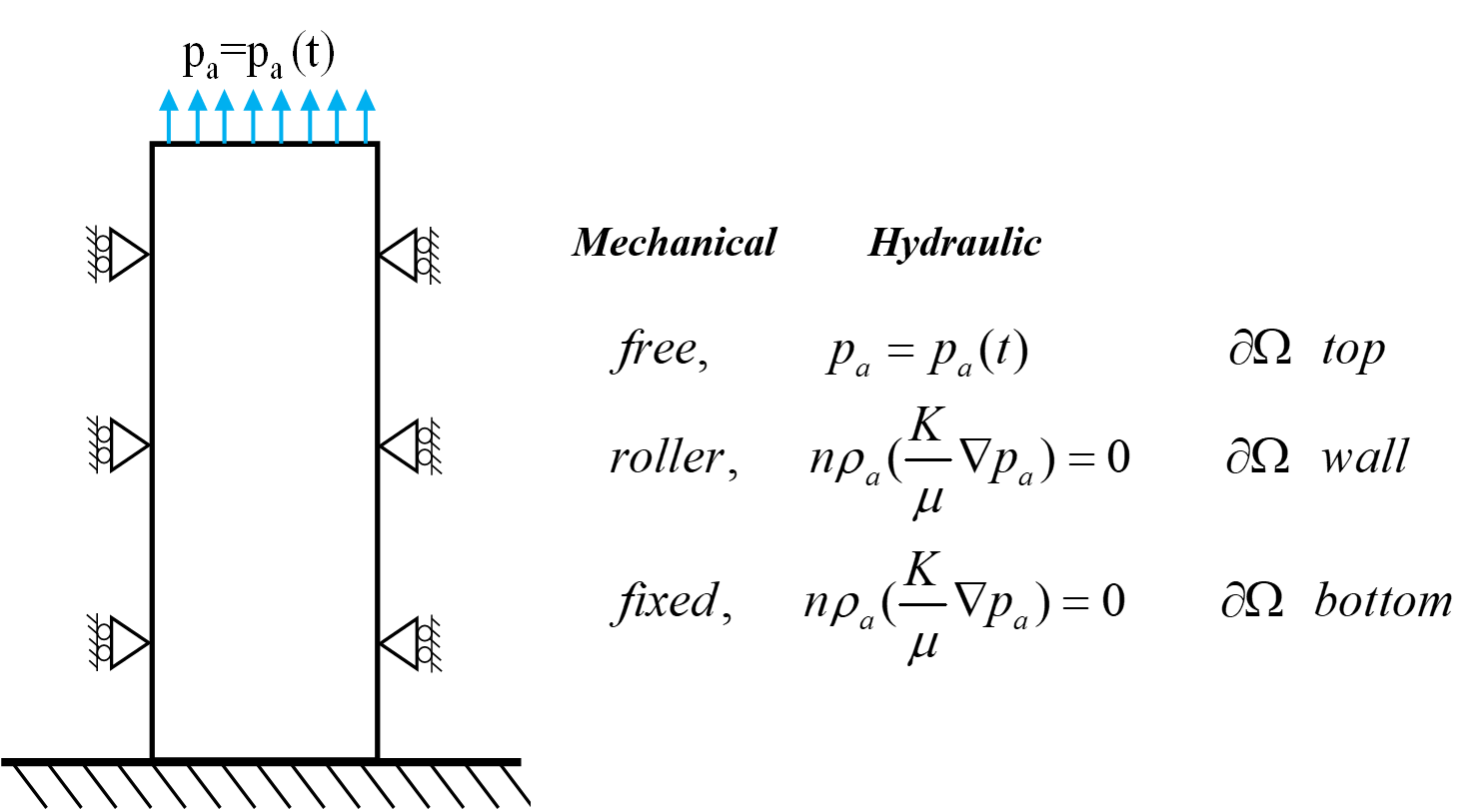


**Supplementary Fig. S2.** Boundary conditions for depressurization simulation model. *P*_a_ (t) is the absolute pressure at the top surface of the specimen and is a function of the time.


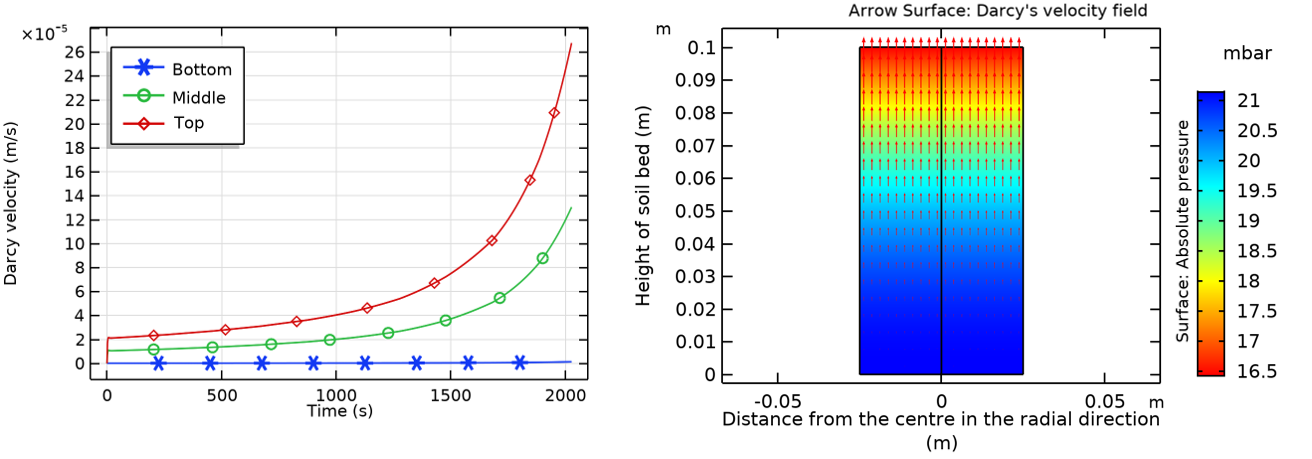


**(a) (b)**

**Supplementary Fig. S3**. Numerical simulation results for the depressurization test on the simulant bed of 0.1 m height and a depressurization rate of 0.5 mbar s^–1^. (a) Darcy velocity (m s^–1^) at three typical locations: top, middle, and bottom of the simulant bed. (b) Darcy velocity field (red arrows) and absolute pressure contour.
